# Machine Learning-Based Radiomics for Prediction of Epidermal Growth Factor Receptor Mutations in Lung Adenocarcinoma

**DOI:** 10.1155/2022/2056837

**Published:** 2022-05-07

**Authors:** Jiameng Lu, Xiaoqing Ji, Lixia Wang, Yunxiu Jiang, Xinyi Liu, Zhenshen Ma, Yafei Ning, Jie Dong, Haiying Peng, Fei Sun, Zihan Guo, Yanbo Ji, Jianping Xing, Yue Lu, Degan Lu

**Affiliations:** ^1^School of Microelectronics, Shandong University, Jinan 250100, China; ^2^Department of Nursing, The First Affiliated Hospital of Shandong First Medical University & Shandong Provincial Qianfoshan Hospital, Jinan 250014, China; ^3^Division of Disinfecting and Supply, Liaocheng People's Hospital, Liaocheng 252000, China; ^4^Graduate School of Shandong First Medical University, Jinan 250000, China; ^5^Department of Radiology, The First Affiliated Hospital of Shandong First Medical University & Shandong Provincial Qianfoshan Hospital, Shandong Medicine and Health Key Laboratory of Abdominal Medicine Imaging, Shandong Lung Cancer Institute, Shandong Institute of Neuroimmunology, Jinan 250000, China; ^6^Department of Interventional Medicine, The Second Hospital, Cheeloo College of Medicine, Shandong University; Interventional Oncology, Institute of Shandong University, Jinan 250033, China; ^7^Department of Respiratory, The First Affiliated Hospital of Shandong First Medical University & Shandong Provincial Qianfoshan Hospital, Shandong Institute of Respiratory Diseases, Shandong Institute of Anesthesia and Respiratory Critical Medicine, Jinan 250000, China

## Abstract

Identifying an epidermal growth factor receptor (EGFR) mutation is important because EGFR tyrosine kinase inhibitors are the first-line treatment of choice for patients with EGFR mutation-positive lung adenocarcinomas (LUAC). This study is aimed at developing and validating a radiomics-based machine learning (ML) approach to identify EGFR mutations in patients with LUAC. We retrospectively collected data from 201 patients with positive EGFR mutation LUAC (140 in the training cohort and 61 in the validation cohort). We extracted 1316 radiomics features from preprocessed CT images and selected 14 radiomics features and 1 clinical feature which were most relevant to mutations through filter method. Subsequently, we built models using 7 ML approaches and established the receiver operating characteristic (ROC) curve to assess the discriminating performance of these models. In terms of predicting EGFR mutation, the model derived from radiomics features and combined models (radiomics features and relevant clinical factors) had an AUC of 0.79 (95% confidence interval (CI): 0.77-0.82), 0.86 (0.87-0.88), respectively. Our study offers a radiomics-based ML model using filter methods to detect the EGFR mutation in patients with LUAC. This convenient and low-cost method may be of help to noninvasively identify patients before obtaining tumor sample for molecule testing.

## 1. Introduction

Lung cancer was the second most commonly diagnosed cancer and remained the leading cause of cancer-related death worldwide [1]. The most common histological subtype of lung cancer is lung adenocarcinoma (LUAC), accounting for approximately 40% of all cases [2]. Although tremendous progress has been made in the treatment of LUAC in the last decade, the prognosis of patients who are detected at advanced clinical stage remains unfavorable. Epidermal growth factor receptor (EGFR) is one of the most frequently mutated genes in LUAC [3], and EGFR tyrosine kinase inhibitors (TKI) have provided patients who harbor activating EGFR mutations with clinical benefit, such as high response rate and prolonged progression-free survival (PFS) [4]. Therefore, an EGFR-TKI has become the first-line treatment of choice for patients with positive EGFR mutation LUAC [5]. As a result, the detection of EGFR mutations is of great significance in determining treatment for patients with LUAC [6].

Detection of EGFR mutational profile is currently based on cytology and noncytology biopsy samples, and mutational sequencing has become the gold standard of EGFR mutation detection [7]. However, tissue sampling has some disadvantages. First, the tumor tissue is not easy to obtain in several cases. Second, the biopsied sample does not necessarily represent the tumor tissue due to intratumor heterogeneity [8]. Third, biopsy testing may potentially increase the risk of cancer metastasis, although the chance is small [9]. Finally, long turnaround time, unfeasibly repeated biopsy, and the relative high costs also account for the limited use of mutational sequencing [10]. Thus, it is a critical need to explore a noninvasive and convenient method to predict EGFR mutation status.

Radiomics is a rapidly evolving and important field because it can extract and analyze multiple features derived from digital medical images with the aim of enhancing clinical decision-making [11, 12]. Studies have revealed that somatic mutations, which ultimately lead to tumor phenotype, can be predicted by radiomics in different solid tumors, including lung cancer [10, 13]. Based on imaging information extracted from magnetic resonance imaging (MRI), computed tomography (CT), and positron-emission-tomography (PET), radiomics analysis can be performed to identify the presence of EGFR, anaplastic lymphoma kinase (ALK), Kirsten rat sarcoma viral oncogene (KRAS), and Erb-B2 receptor tyrosine kinase 2 (ERBB2) mutations in patients with non-small-cell lung cancer (NSCLC) [14–18]. With specific regard to EGFR mutation, previous studies have documented the potential for radiomics to predict EGFR 19Del and L858R based on the phenotypic appearance [14, 16, 19]. For example, Rossi et al. built a machine learning (ML) model to identify EGFR mutant and achieved an area under the receiver operating characteristic curve (AUC) of 0.89 [19]. By developing deep learning models, Zhang et al. reported that radiomics features from CT images can discriminate EGFR mutation with an AUC of 0.910 and 0.841 for the internal and external test cohorts, respectively [20]. Hong and colleagues [21] utilized features from enhanced CT imaging to recognize EGFR mutation status in advanced LUAC. They reported an AUC of 0.851 for predicting EGFR mutation with a model based on radiomics features and clinical data [21]. Although previous studies have documented the association between radiological characteristics and EGFR mutation status, the role of CT-based radiomics ML in identifying EGFR mutation in LUAC remains to be further explored.

Selection of a subset of relevant predictor variables from highly dimensional data, which is termed as feature selection (FS), is a critical step in analysis of radiomics features [22]. FS is the core of classification which plays a fundamental role in ML and can reduce the learning complexity. As one of the FS methods, filter methods assess the goodness of features based on a simple weight score criterion [23]. In addition, filter methods select features independent of any specific classifiers and demand less computation [23]. As a result, filter models have been widely studied because of their efficiency and simplicity. However, few studies on prediction of EGFR mutation status were reported using filter approaches based on ML.

Therefore, the aim of this study is to develop a radiomics-based model to predict EGFR mutation status in patients with LUAC using filter methods. In the present study, CT-based radiomics features and ML methods were used to identify EGFR mutation status and the effect of this model on predicting EGFR mutation in LUAC was assessed. The outcome of this study may aid in distinguishing patients with EGFR mutations from those without and helping clinicians to make treatment decisions for patients.

## 2. Materials and Methods

### 2.1. Patients

**Figure 1:**
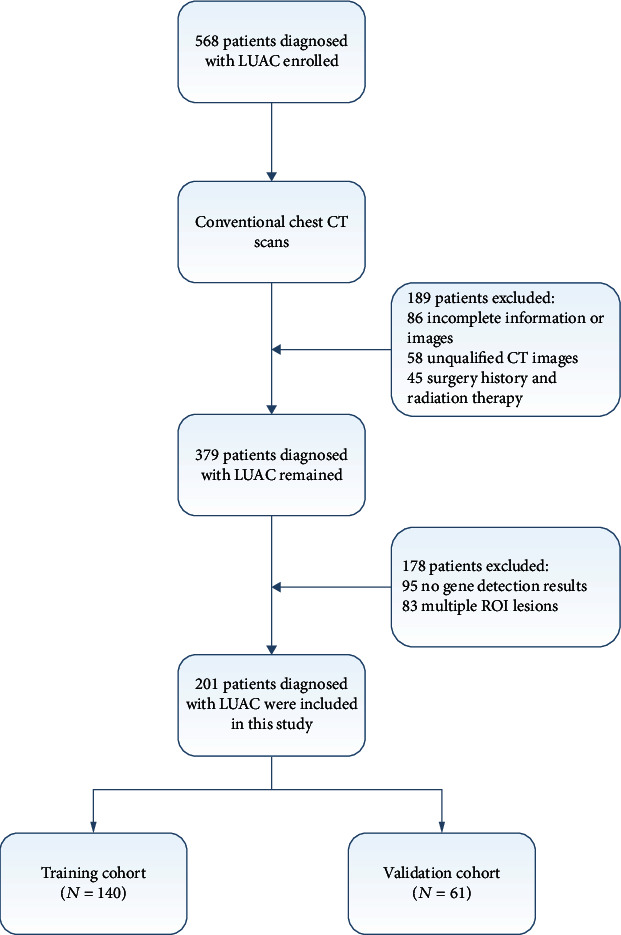
Patient recruitment workflow. In total, 201 of 568 patients were included in this study according to the selection criteria.

**Figure 2:**
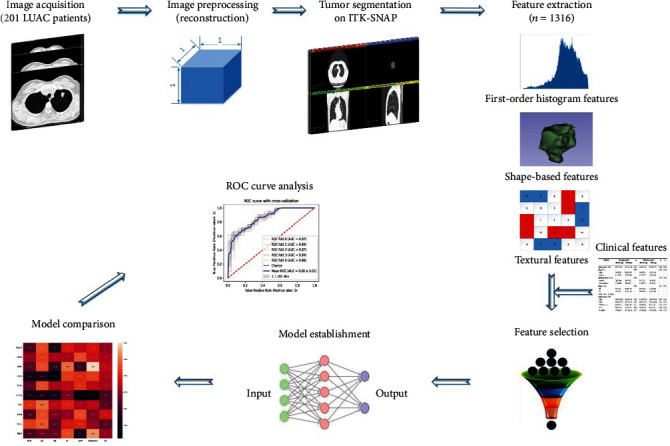
Workflow of the radiomics analysis.

The study population was retrospectively selected from patients diagnosed with LUAC from the First Affiliated Hospital of Shandong First Medical University (Jinan, China). The institutional review board approved this study with a waiver for the informed consent requirement. Patients who were (1) histologically diagnosed with primary LUAC, (2) classified as stage III-IV according to the Eighth Edition of the Lung Cancer Stage Classification, (3) having detected EGFR mutations based on PCR technology, (4) treatment-naïve subjects, and (5) receiving chest CT scan prior to biopsies or surgery met the inclusion criteria and were included. The exclusion criteria were given as follows: (1) lack of clinical data, such as age, gender, stage, and serum tumor marker, and (2) difficulty in drawing regions of interest (ROIs). In the end, 201 patients were included in this study. The flow chart of participant recruitment is shown in Figure 1. The enrolled patients were randomly classified into the training cohort and independent validation cohort with the ratio of 7 (*n* = 140) : 3 (*n* = 61). The workflow of the radiomics analysis is depicted in Figure 2.

### 2.2. Analysis of EGFR Mutation

Based on the tumor specimen, EGFR gene mutations in exons 18, 19, 20, and 21 were examined by an amplification refractory mutation system real-time technology using Human EGFR Gene Mutations Fluorescence Polymerase Chain Reaction (PCR) Diagnostic Kit (Amoy Diagnostics Co., Ltd, Xiamen, China). Wild-type EGFR in the present study referred to absence of mutation on those loci.

### 2.3. Image Acquisition

All patients included in this study underwent chest CT scans prior to any treatment using two CT scanners (GE Healthcare, Milwaukee, WI, USA; United Imaging, Shanghai, China). The scanning parameters were given as follows: the tube voltage, 120 kVp; tube current, 160–300 mA; detector collimation, 64 or 128 × 0.625 mm; field of view, 350 × 350 mm; the pitch, 0.992 : 1; and matrix of 512 × 512. All images were reconstructed with a section thickness of 2 mm and were stored in DICOM format in the Picture Archiving and Communication Systems (PACS) of our hospital.

### 2.4. Image Preprocessing

Because different CT scans were used in this study, image preprocessing prior to segmentation and feature extraction was undergone to make the radiomics features more robust [24]. As previously reported by Hong et al. [21], a resampling method and Gaussian filter were used in this process.

### 2.5. Tumor Segmentation

Every lesion was independently evaluated and segmented manually slice by slice by two senior radiologists (both with more than 10-year experience of CT interpretation). The ROI was delineated in ITK-SNAP (version 3.6, http://www.itksnap.org) and confirmed by another chest radiologist with 15-year experience [25, 26]. If one patient has multiple lesions, the radiologist only delineates the tumor area where the biopsy was performed. All radiologists were blinded to the status of EGFR mutation.

To reduce the differences in manual segmentation between two radiologists, the intragroup correlation coefficient (ICC) for each feature was calculated [27, 28]. Only those with an ICC greater than 0.85 was considered highly stable and selected for the following analysis.

### 2.6. Feature Extraction

Based on the three-dimensional region of interest (3D ROI), radiomics features were extracted from each ROI using Pyradiomics package (http://pyradiomics.readthedocs. io/en/latest/index.html). A total of 1316 features were extracted, and these features can be divided into 3 categories: first-order statistics (*n* = 18 features), shape-based (*n* = 14 features), and textural feature [18]. The textural feature category includes Gray-Level Cooccurrence Matrix (GLCM) (*n* = 24 features), Gray-Level Run Length Matrix (GLRLM) (*n* = 16 features), Gray-Level Size Zone Matrix (GLSZM) (*n* = 16 features), Gray-Level Dependence Matrix (GLDM) (*n* = 14 features), and Neighboring Gray Tone Difference Matrix (NGTDM) (*n* = 5 features). In addition, two filters (including wavelet (*n* = 744 features) and Laplacian of Gaussian (*n* = 465 features) were also applied to the original CT images to obtain transformed images. By decomposing the image with wavelet transform, high- (H) or low- (L) pass filters in three dimensions were applied and 8 kinds of combinations were obtained: LHL, HHL, HLL, HHH, HLH, LHH, LLH, and LLL. To emphasize areas of gray-level change, the LoG filter was applied to the input image and yield a derived image for each sigma value specified [29]. In our study, five filters with different sigma values were applied (sigma = 1.0 mm, 2.0 mm, 3.0 mm, 4.0 mm, and 5.0 mm). The specific number of features is listed in supplementary Table 1.

### 2.7. Feature Selection

At first, univariate analysis was performed for each feature and those with *P* values < 0.1 were considered to be associated with genetic mutations and selected [30]. Then, 10 FS techniques based on filter methods were used in the current analysis and they can be classified into two categories: univariate methods and multivariate methods [31]. The univariate methods included Fisher score (FSCR), Relief (RELF), *t*-test score (TTSC), chi-square (CHSQ), Wilcoxon rank sum (WLCX), Gini index (GINI), information gain (IFGN), *F*-ANOVA (FAOV), and Pearson correlation coefficient (PESC). The multivariate methods consisted of mutual information (MUIF). These approaches were chosen mainly due to their computational efficiency, simplicity in implementation, and applications in literature [32, 33]. Filter methods calculate a relevance score for each feature, and those which are lower than a given threshold will be removed [31].

FS methods, such as GINI, RELF, and IFGN, were performed using the “attrEval” function from the “CORElearn” package in R software package. FAOV, FSCR, TTSC, CHSQ, WLCX, PESC, and MUIF were implemented using the scikit-learn package in Python software (Python Software Foundation: http://www.python.org). In order to describe various aspects of the EGFR mutation and avoid choosing features from a certain feature group, features were selected based on rankings in their own group rather than rankings among all features. With increased numbers of selected features, we found that the majority of classifiers showed the best predictive performance when the top 2 features are selected from each group. If no features passed the univariate test in a certain group, this group will be ignored.

### 2.8. Radiomics Model Establishment and Performance Evaluation

Seven ML algorithms were imported from the scikit-learn library in Python software to establish models [34]. These algorithms included decision tree (DT), AdaBoost classifier (AD), naïve Bayes (NB), random forest (RF), logistic regression (LR), support vector machines (SVM), extreme gradient boosting (XGBoost, XGB), and *k* nearest neighbors (KNN). In combination of 10 FS methods and 7 classifiers, we developed 70 (10 × 7 = 70) models. The nomenclature of each model was established by two elements: the name of FS method and classifier. For example, NB-WLCX referred to a model combining naïve Bayes classifier with FS approach of Wilcoxon rank sum. The predictive ability of each algorithm was primarily assessed using AUC of receiver operating characteristic (ROC) curve analysis. Then, fivefold cross-validation was applied to examine all results and also evaluated by AUC. The model which gives the highest cross-validation accuracy was selected as the final model for further analysis.

### 2.9. Development and Validation of Models Combining Radiomics Features and Clinical Characteristics

To further increase the power of predicting EGFR mutation, some clinical characteristics were added to the aforementioned model. These clinical factors consisted of age, gender, smoking status, stage of disease, and serum level of tumor markers. The tumor markers included carcinoembryonic antigen (CEA), neuron-specific enolase (NSE), fragment of cytokeratin subunit 19 (CYFRA 21-1), squamous cell carcinoma antigen (SCC), and pro-gastrin-releasing peptide (Pro-GRP). The predictive performance of each algorithm was also evaluated based on the AUC of ROC curve analysis.

### 2.10. Statistical Analysis

Statistical analysis was performed using PRISM version 6 (GraphPad, La Jolla, CA, USA). Quantitative data were compared using Student's *t*-test, and categorical data were compared using the *χ*^2^ test to identify baseline differences. The discrimination performance of models was evaluated by the ROC curve. All statistical tests were two-tailed, and *P* < 0.05 was considered statistically significant.

## 3. Result

### 3.1. Clinical Characteristics

The baseline clinical characteristics of the enrolled patients are listed in Table 1. No evident differences were found among the age, gender, stage of disease, and serum level of CEA, NSE, CYFRA 21-1, and Pro-GRP between the EGFR-mutated and EGFR wild-type group (*P* > 0.05). The smoking status was significantly different between the EGFR-mutated and EGFR wild-type group in the training cohort (*P* < 0.05). The level of SCC in the serum was significantly different in the training and validation set (*P* < 0.05).

### 3.2. Selected Stable Features

In total, 1316 radiomics features were extracted. Subsequently, ICC for radiomics features in each group were calculated (ICC = mean ± SD) and are depicted in supplementary Fig. 1: shape-based features (ICC = 0.97 ± 0.03), first-order features (ICC = 0.98 ± 0.01), GLCM features (ICC = 0.98 ± 0.02), GLRLM features (ICC = 0.99 ± 0.01), GLSZM features (ICC = 0.98 ± 0.02), GLDM features (ICC = 0.98 ± 0.01), NGTDM features (ICC = 0.99 ± 0.01), wavelet transformed features (ICC = 0.97 ± 0.05), and LoG-transformed features (ICC = 0.95 ± 0.06). Overall, 1269 of the 1316 (96.4%) extracted radiomics features were identified as stability and were retained. These features consist of 14 shape-based features, 18 first-order features, 24 GLCM features, 16 GLRLM features, 16 GLSZM features, 14 GLDM features, 49 LoG features, 5 NGTDM features, 727 wavelet transformed features, and 435 LoG-transformed features. The histogram of the ICC values of the radiomics features is shown in supplementary Figure 1.

### 3.3. Model Performance Assessment

**Figure 3:**
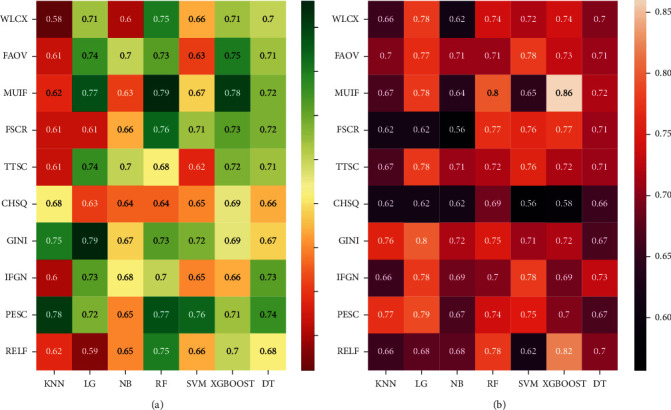
Heat maps with the AUC of different combinations of FS methods (rows) and classification algorithms (columns). (a) The average cross-validated AUC from 70 models based on radiomics features. (b) The average cross-validated AUC from 70 models based on radiomics features and clinical data.

**Figure 4:**
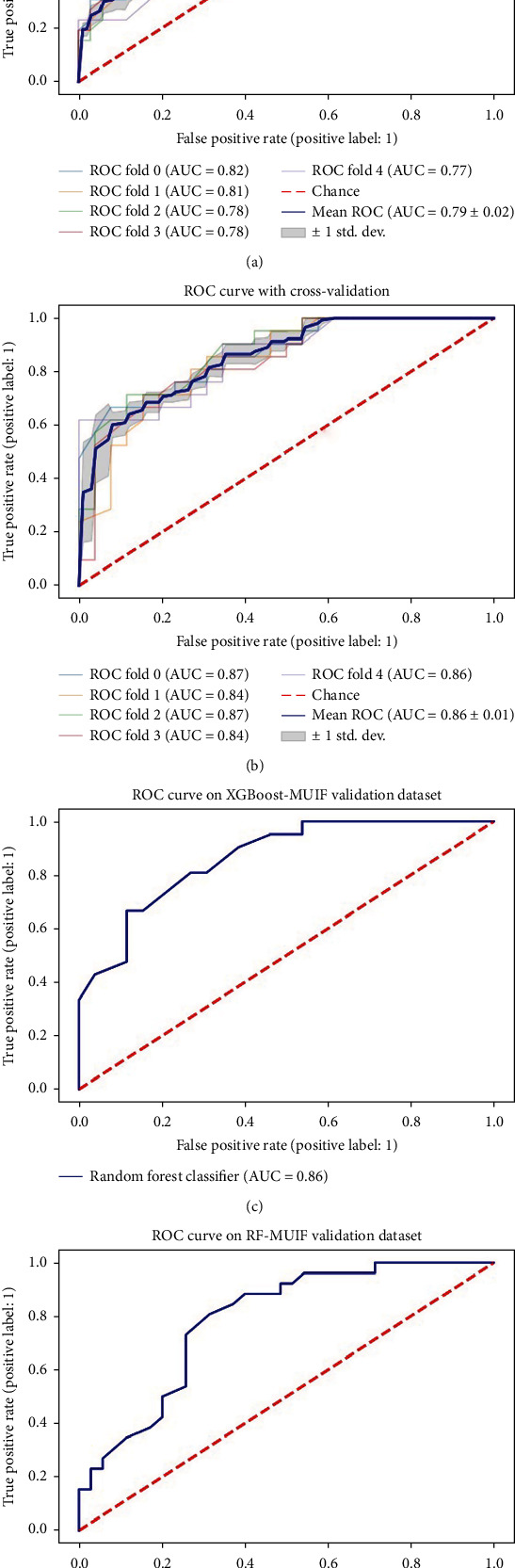
ROC curves of models on the training and validation sets. (a) The fivefold cross-validated ROC curve of model RF-MUIF. (b) The fivefold cross-validated ROC curve of model XGBoost-MUIF. (c) ROC curve of XGBoost-MUIF on the validation dataset. (d) ROC curve of RF-MUIF on the validation dataset.

The mean AUC scores for each classifier across the different FS methods are presented in a heat map form (Figure 3). When analysis was based on radiomics features, the RF classifier performed better than the other classifiers and the median AUC of the 10 models using RF classifier was 0.74. With regard to FS approaches, MUIF provided the best predictive performance and the median AUC of the 7 models using MUIF FS method was 0.72. When various classifiers and FS methods are combined, RF-MUIF model provided the highest performance in the prediction of EGFR mutation and the AUC reached 0.79 (Figure 3(a)). Moreover, the RF-MUIF model achieved a sensitivity of 0.81, a specificity of 0.63, and an accuracy of 0.74 for predicting EGFR mutation status. Further, the XGBoost model outperformed other classifiers (median AUC 0.73) and MUIF generated better AUCs (median AUC 0.72) when the integrated model built with radiomics signature and clinical features was analyzed. The model of XGBoost-MUIF achieved the best predictive performance, and the AUC, sensitivity, specificity, and accuracy were 0.86, 0.95, 0.72, and 0.83, respectively (Figure 3(b)). The cross-validated AUC scores and AUC curve on the validation dataset are shown in (Figures 4(a)–4(d)).

### 3.4. Analysis of the Selected Radiomics and Clinical Features

**Figure 5:**
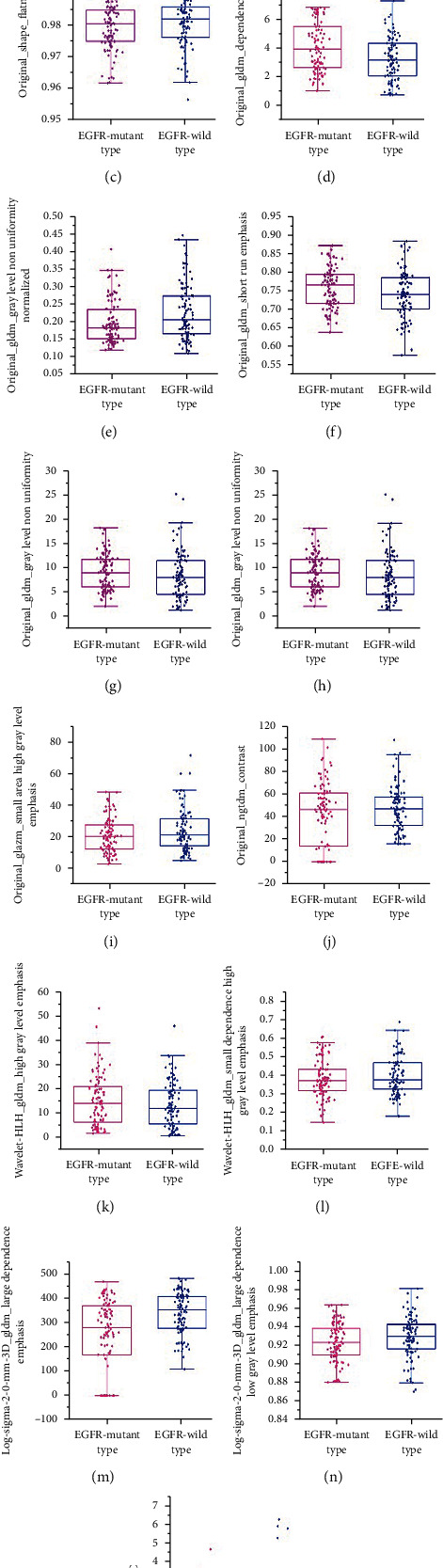
Boxplot illustrating the variation of the 15 features that finally incorporate into XGBoost-MUIF model between EGFR mutant type and EGFR wild type: (a) original_shape_Flatness; (b) original_firstorder_Kurtosis; (c) original_glcm_Idmn; (d) original_gldm_DependenceEntropy; (e) original_glrlm_GrayLevelNonUniformityNormalized; (f) original_glrlm_ShortRunEmphasis; (g) original_gldm_GrayLevelNonUniformity; (h) original_glszm_LargeAreaLowGrayLevelEmphasis; (i) original_glszm_SmallAreaHighGrayLevelEmphasis; (j) original_ngtdm_Contrast; (k) wavelet-HLH_gldm_HighGrayLevelEmphasis; (l) wavelet-HLH_gldm_SmallDependenceHighGrayLevelEmphasis; (m) log-sigma-2-0-mm-3D_gldm_LargeDependenceEmphasis; (n) log-sigma-2-0-mm-3D_gldm_LargeDependenceLowGrayLevelEmphasis; (o) SCC.

Among the selected radiomics and clinical features, 10 features had lower values for EGFR mutant type than for EGFR wild type. These features included original_shape_Flatness (0.57 vs. 0.60, *P* = 0.09), original_firstorder_Kurtosis (5.47 vs. 8.12, *P* = 0.004), original_glrlm_GrayLevelNonUniformityNormalized (0.20 vs. 0.23, *P* = 0.008), original_ngtdm_Contrast(41.36 vs. 46.74, *P* < 0.001), wavelet-HLH_gldm_SmallDependenceHighGrayLevelEmphasis (0.38 vs. 0.40, *P* < 0.001) log-sigma-2-0-mm-3D_gldm_LargeDependenceEmphasis (247.74 vs. 338.90, *P* < 0.001), log-sigma-2-0-mm-3D_gldm_LargeDependenceLowGrayLevelEmphasis (0.91 vs 0.93, *P* < 0.001), original_glcm_Idmn (0.97 vs. 0.99, *P* = 0.09), original_glszm_SmallAreaHighGrayLevelEmphasis (20.63 vs. 23.87, *P* = 0.06), and SCC (3.91 vs. 6.03, *P* = 0.15). Five features showed higher value for EGFR mutant type compared with EGFR wild type. These features consisted of original_glrlm_ShortRunEmphasis (0.76 vs. 0.73, *P* = 0.03), original_glszm_LargeAreaLowGrayLevelEmphasis (2.36 vs. 2.20, *P* = 0.07), original_gldm_DependenceEntropy (8.40 vs. 5.84, *P* < 0.001), original_gldm_GrayLevelNonUniformity (9.11 vs. 8.44, *P* < 0.001), and wavelet-HLH_gldm_HighGrayLevelEmphasis (15.18 vs 13.56, *P* < 0.001) (Figure 5).

## 4. Discussion

In this retrospective study, we proposed a stable predictive model based on noninvasive CT images and clinical features in order to predict EGFR mutation status for patients with LUAC. The ML model was trained with 140 patients, and its performance was validated with 61 patients. This model showed favorable predictability in the validation set (AUC = 0.79). Similarly, the AUC of the integrated model built with radiomics features and clinical data was 0.86. This study demonstrated that the association was evident between CT image features and EGFR genotype and the ability of radiomics to identify the EGFR mutation status. Therefore, it is possible to predict EGFR mutation before invasive biopsy and expensive molecular testing based on a noninvasive method. To the best of our knowledge, this is the only study which establishes ML models using filter methods to predict EGFR mutation status in patients of LUAC. The present study has made new contributions to the existing research in this field.

Radiomics is defined as the extraction of a myriad of radiographic image features and the further mining of these data with the intent of supporting adoption of precision medicine [35]. Radiomics analysis can be used to increase precision in establishing a diagnosis, assessing prognosis, and predicting therapy response in cancer patients. Some features have even been shown to identify genomic alterations in tumor tissue, which is termed as “radiogenomics” [36]. Radiogenomics examines the relationship between disease genomic characteristics and its radiomics features [37]. Although some limitations of the radiogenomics approach exist, radiogenomics will play an important role in cancer research because it paves an avenue of obtaining important information from limited and incomplete data. This information might improve decision-making and, as a result, leads to better patient outcomes [38]. For example, recent studies have shown that radiogenomics can aid in treatment option and prognosis assessment in NSCLC patients [39, 40]. Additionally, radiogenomics can help in evaluating the efficacy of therapy and predicting outcomes of treatment [37, 39].

Previous studies have shown that EGFR mutation status can be predicted from image features in patients with NSCLC. For example, a study by Zhang et al. found that radiomics features are able to discriminate EGFR mutation in patients with NSCLC and the AUC was 0.862 and 0.873 for the training and validation cohort, respectively [41]. Mei et al. [42] analyzed the association between CT texture features and EGFR mutation statuses in patients with LUAC. They reported that AUC of combination with clinical and radiomics features to predict EGFR mutations was 0.664. Liu et al. [43] also predict EGFR status with a model based on five radiomics features and obtain an AUC of 0.647 in surgically resected peripheral LUAC. When combined with clinical data, this model can reach an AUC of 0.709. In the study conducted by Gevaert et al. [44], the authors built a predictive model for the EGFR mutation and achieved an AUC of 0.89. Their work showed the potential of semantic image features to predict molecular properties. Recently, Wang et al. proposed a deep learning model to distinguish EGFR mutation status using CT images and clinical data. The AUC was 0.85 and 0.81 in the training and test cohorts, respectively [16]. Our results combined with previous studies clearly demonstrate that radiogenomics powered by ML can potentially aid in identifying patients who will benefit from targeted therapy.

FS is a process often used in ML, wherein a subset of predictor variables is selected from the input data for application of a learning algorithm [23]. FS is the core of classification which plays an essential role in image processing and ML [22]. The aims of FS include, but are not limited to, the following aspects: preventing overfitting of predictive and classifier models and achieving a good prediction performance, providing quicker and more optimizing computational solutions, and gaining a better insight into the underlying processes by which the data are generated [31, 32]. FS methods usually consist of three categories: wrapper, embedded, and filter. Most wrapper approaches are not computationally feasible for high-dimensional data sets [32]. Embedded methods search for the most optimal features during the training of the classifier, and they have better computational complexity than wrapper methods [45]. Filter methods calculate a score for each predictor variable and select those which exceed a defined threshold [31]. Unlike wrapper and embedded methods which are specific to a given learning algorithm, filter methods could be combined with any kind of predictive approaches [31]. Due to its independence of learning algorithms, filter approaches can prevent overfitting and demand less work in computation than wrapper and embedded methods [31]. As a result, although filter-based feature selection methods have some shortcomings, such as ignoring feature dependencies and providing feature subsets which perhaps contain redundant information, filter methods are increasingly used due to their efficiency, simplicity, and a good generalization capacity [46]. Zhang et al. built ML models based on CT radiomics features which were selected using filter methods to discriminate arteriovenous malformation-related intraparenchymal hematomas from those that were associated with other etiologies [47]. They obtained AUCs of 0.988 and 0.957 in the training and test cohorts, respectively. In the work presented by Parmar et al. [33], the authors showed that choosing WLCX, one of the filter methods, and/or RF classification method gets the highest performance in survival prediction based on 440 radiomics features extracted from 464 lung cancer patients. Our models achieved an AUC of 0.79 to identify EGFR mutation, which is comparable to the previous reports. It is worth noting that a deep learning approach has some shortcoming: requiring a huge amount of data for training, relying on more specialized hardware and computing power, and lack of interpretability [48, 49].

As a branch of artificial intelligence, ML is a method to identify patterns and relationships in data by building algorithmic models. ML has also been proven to be an interesting field in biomedical research and focuses on teaching computers to perform classification, prediction, or estimation and improve its own performance based on some experience (data) [50]. Supervised learning (training data are labeled) and unsupervised learning (training data are unlabeled) are two main common types of ML methods, and the former has been a dominant method in the data mining field [51]. Our retrospective study showed that it was feasible for 7 ML approaches to predict EGFR mutation status. When used in combination with the RF classifier, the majority of FS methods achieved the best predictive performance. This finding is in accordance with a recently reported study by Parmar et al. [33], who found that RF classification method yields the highest performance in the prediction of two-year patient survival in NSCLC patients. Gu et al. reported that RF-based radiomics classifier performed best (AUC = 0.776) in predicting the Ki-67 expression level in NSCLC [52]. Uddin et al. [51] compared different types of supervised ML algorithms to evaluate the potential for disease risk prediction. They found that the SVM algorithm is most frequently used whereas the RF algorithm gave superior accuracy comparatively. In addition, MUIF was found to have the highest predictive power with the majority of classifiers. MUIF can be used as relevant criterion for selecting predictive subsets of features [53]. Under some reasonable assumptions, features selected with MUIF are those whose mean squared error and mean absolute error are minimizing [54]. Our results combined with previous researches demonstrate that RF together with MUIF is a better ML approach for identifying EGFR mutations based on radiomics features.

The potential clinical utility of radiomics based model has also been assessed to predict EFGR mutation in this study. We identified SCC as the most important clinical predictor, which was consistent with previous reports [55, 56]. We found that age, gender, and s-CEA were not associated with the EGFR mutation status, which did not accord with previous studies [21, 57–59]. A meta-analysis of human epidemiologic data revealed that there are significantly increased odds of EGFR mutation in never smokers in comparison to ever smokers [60]. Hong et al. reported that female was more likely (OR = 3.124) to have EGFR mutations [21]. Wang et al. [57] demonstrated that high preoperative serum CEA levels (CEA > 20 ng/mL) were effective for predicting the EGFR mutation. With regard to the models integrating clinical characteristics and radiomics features, we found that the XGBoost-MUIF model performed better in predicting EGFR mutation status. These results are consistent with a previous study that reported that the genetic algorithm plus XGBoost classifier had the most favorable performance and reached an accuracy of 0.836 for detecting EGFR in patients with NSCLC [61].

The present study has some limitations. First, as the study was retrospective in nature, it was associated with flaws such as possible information and selection bias. Second, our sample size is relatively small. However, although larger data sets are associated with more power, radiomics analyses can be performed with as few as 100 patients [62]. Further studies on large sample are required to assess the clinical applications as well as the stability of our models. Third, there were differences in the prevalence of EGFR mutations in LUAC and in subsequent treatments among different races [63], but all of subjects who were involved in this study were Chinese. Therefore, the results may lack universality and needs further verification within other racial and ethnic population. Finally, manual segmentation of ROI is time-consuming and its reproducibility should be evaluated by interobserver reproducibility analysis. Semiautomated or automated radiomics methods are expected in our future research to improve the robustness.

## 5. Conclusions

In conclusion, the present study showed that radiomics signature extracted from CT images in combination with clinical characters can identify EGFR mutation status in LUAC. Although these findings remain to be validated with a larger sample size, ML-based radiomics using filter methods provides a noninvasive and low-cost method to predict EGFR mutations, which may aid in screening patients before invasive sampling and developing personalized treatment design for optimizing the outcomes of patients with LUAC.

## Figures and Tables

**Table 1:** Characteristics of patients in training and validation cohorts.

Variable	Training cohort		*P*	Validation cohort		*P*	*P*
	Mutant type	Wild type		Mutant type	Wild type		
Age (y, mean ± SD)	65.27 ± 1.44	66.14 ± 1.24	0.66	64.35 ± 1.34	63.48 ± 1.57	0.68	0.83
Sex, *n* (%)			0.06			0.19	0.44
Male	34 (24.29)	40 (28.57)		9 (14.75)	19 (31.15)		
Female	41 (29.29)	25 (17.86)		17 (27.87)	16 (26.23)		
Smoking status, *n* (%)			0.01			0.05	0.64
Smoker	24 (32.00)	36 (55.38)		5 (19.23)	19 (54.29)		
Never smoker	51 (68.00)	29 (44.62)		21 (80.77)	16 (45.71)		
Stage, *n* (%)			0.43			0.15	0.07
III	46 (61.33)	44 (68.75)		16 (61.54)	15 (42.86)		
IV	29 (38.67)	20 (31.25)		10 (38.46)	20 (57.14)		
Serum level of tumor marker (mean ± SD)							
CEA	109.0 ± 75.82	30.86 ± 7.56	0.31	114.6 ± 77.44	129.3 ± 78.20	0.89	0.28
NSE	98.39 ± 75.75	28.75 ± 6.79	0.36	100.6 ± 77.31	29.05 ± 6.932	0.36	0.27
CYFRA 21-1	6.91 ± 0.79	9.36 ± 1.58	0.17	6.88 ± 0.82	9.63 ± 1.62	0.13	0.17
SCC	0.85 ± 0.77	1.26 ± 1.65	0.08	0.62 ± 0.38	0.97 ± 0.92	0.06	0.03
Pro-GRP	45.50 ± 8.23	49.31 ± 6.49	0.72	45.33 ± 8.40	51.17 ± 7.09	0.59	0.72

CEA: carcinoembryonic antigen; NSE: neuron-specific enolase; CYFRA 21-1: fragment of cytokeratin subunit 19; SCC: squamous cell carcinoma antigen; Pro-GRP: pro-gastrin-releasing peptide.

## Data Availability

The original data supporting the conclusions of this paper will be provided unreservedly by the authors to any qualified researcher.

## Supplementary Materials

Supplementary Materials Table S1: the category and number of features. Figure S1: histogram of the ICC for radiomics features. ICC: intragroup correlation coefficient.
