# Supplementary material for: Machine Learning-Based Radiomics for Prediction of Epidermal Growth Factor Receptor Mutations in Lung Adenocarcinoma
Source: Dis Markers. 2022 May 7;2022:2056837. doi: 10.1155/2022/2056837 (PMC9107363; doi:10.1155/2022/2056837)
Supplement: Supplementary Materials — Table S1: the category and number of features. Figure S1: histogram of the ICC for radiomics features. ICC: intragroup correlation coefficient. [file 2056837.f1.zip › 2056837.f1/Table S1.docx]

**Supplementary Table 1.** The category and number of features

|  |  | **Shape based feature** | **First Order feature** | **GLCM**  **Feature** | **GLDM Feature** | **GLRLM Feature** | **GLSZM**  **Feature** | **NGTDM**  **Feature** | **Total** |
| --- | --- | --- | --- | --- | --- | --- | --- | --- | --- |
| Original images | | 14 | 18 | 24 | 14 | 16 | 16 | 5 | 107 |
| Wavelet transformed  images | LLH |  | 18 | 24 | 14 | 16 | 16 | 5 | 93 |
|  | LHL |  | 18 | 24 | 14 | 16 | 16 | 5 | 93 |
|  | LHH |  | 18 | 24 | 14 | 16 | 16 | 5 | 93 |
|  | HLL |  | 18 | 24 | 14 | 16 | 16 | 5 | 93 |
|  | HLH |  | 18 | 24 | 14 | 16 | 16 | 5 | 93 |
|  | HHL |  | 18 | 24 | 14 | 16 | 16 | 5 | 93 |
|  | HHH |  | 18 | 24 | 14 | 16 | 16 | 5 | 93 |
|  | LLL |  | 18 | 24 | 14 | 16 | 16 | 5 | 93 |
| LoG  transformed  images | Sigma  =1mm |  | 18 | 24 | 14 | 16 | 16 | 5 | 93 |
|  | Sigma  =2mm |  | 18 | 24 | 14 | 16 | 16 | 5 | 93 |
|  | Sigma  =3mm |  | 18 | 24 | 14 | 16 | 16 | 5 | 93 |
|  | Sigma  =4mm |  | 18 | 24 | 14 | 16 | 16 | 5 | 93 |
|  | Sigma  =5mm |  | 18 | 24 | 14 | 16 | 16 | 5 | 93 |
|  |  |  |  |  |  |  |  |  | 1316 |

GLCM: grey-level co-occurrence matrix

GLDM: gray level dependence matrix

GLRLM: grey-level run-length matrix

GLSZM: gray level size zone matrix

NGTDM: neighbouring gray-tone difference matrix

LoG: Laplacian of Gaussian
