# Supplementary figures and images for: Machine Learning-Based Radiomics for Prediction of Epidermal Growth Factor Receptor Mutations in Lung Adenocarcinoma
Source: Dis Markers. 2022 May 7;2022:2056837. doi: 10.1155/2022/2056837 (PMC9107363; doi:10.1155/2022/2056837)

# Radiomic Features Stability

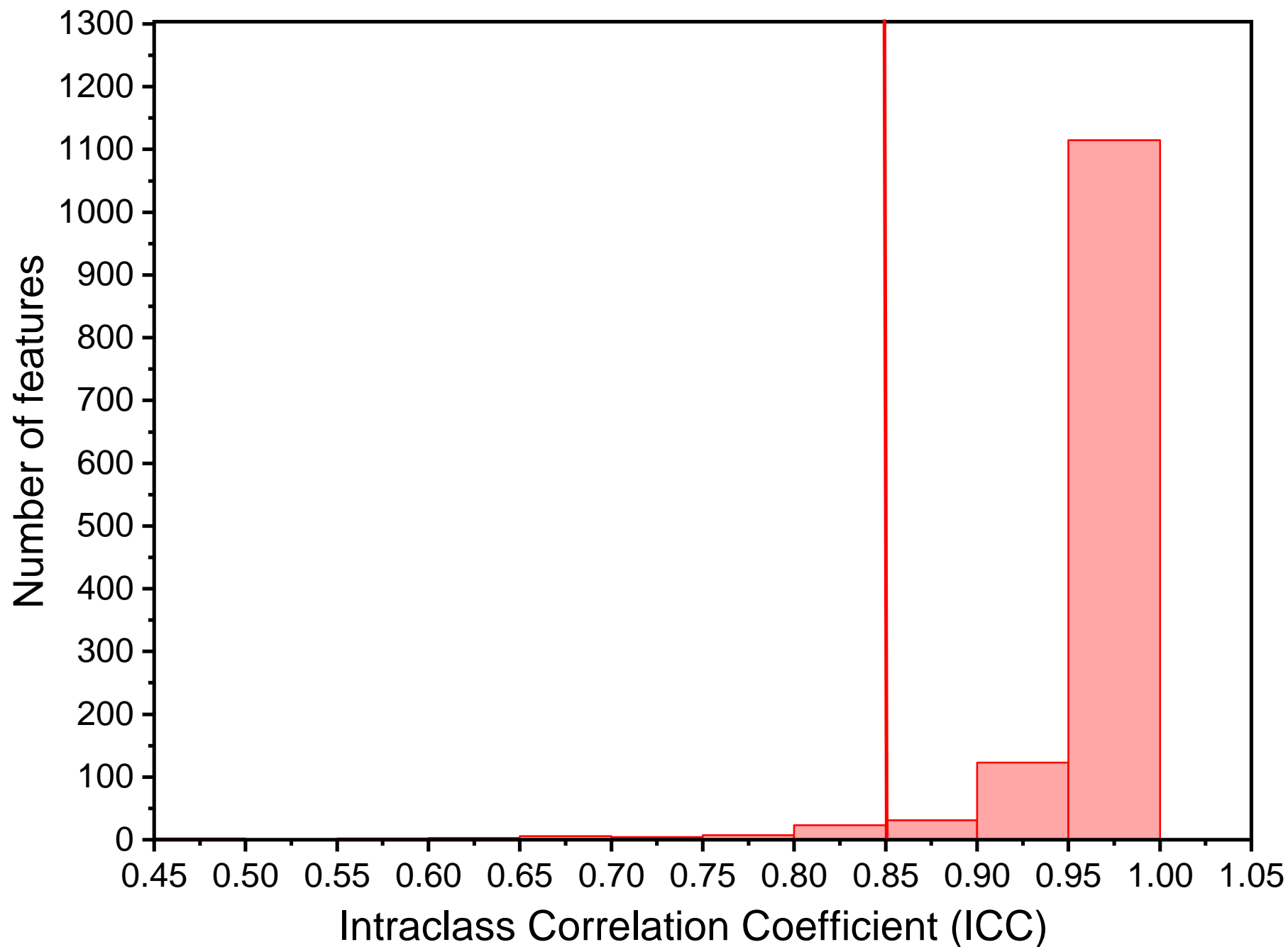

Supplement: Supplementary Materials — Table S1: the category and number of features. Figure S1: histogram of the ICC for radiomics features. ICC: intragroup correlation coefficient. [file 2056837.f1.zip › 2056837.f1/Figure S1.pdf]
